# Impact of Four Ovine *TMEM154* Haplotypes on Ewes during Multiyear Lentivirus Exposure

**DOI:** 10.3390/ijms232314966

**Published:** 2022-11-29

**Authors:** Brad A. Freking, Tom W. Murphy, Carol G. Chitko-McKown, Aspen M. Workman, Michael P. Heaton

**Affiliations:** USDA, ARS, Roman L. Hruska U.S. Meat Animal Research Center, Clay Center, NE 68933, USA

**Keywords:** sheep, *TMEM154* diplotypes, productivity, susceptibility

## Abstract

Polypeptide variation encoded by the ovine transmembrane protein 154 gene (*TMEM154*) is associated with susceptibility to ovine lentivirus, the causative agent of Ovine Progressive Pneumonia (OPP) and Visna/Maedi. Our aim was to compare the four most prevalent *TMEM154* haplotypes on the incidence of infection and ewe productivity during natural multiyear virus exposure. Prospective cohort studies were designed to test gene action and estimate effects of *TMEM154* haplotypes encoding distinctive variant residues: K35 (“1”), I70 (“2”), ancestral (“3”), and A4del/M44 (“4”). Exposure consisted of co-mingling infected ewes at a rate greater than 30% with serological status evaluated every four months. For ewes with one or two copies of the highly susceptible haplotypes “2” and ”3”, the infection prevalence steadily increased to nearly 100% at 55 months. Haplotypes “2” and “3” were equally susceptible and dominant to haplotype “1”. A difference was not detected (*p* < 0.53) in the magnitude of effect with haplotype combinations of “1” and ”4”. The ewe infection prevalence with “1,1”; “1,4”; and “4,4” was 10% to 40% at 55 months. The latter suggested that two copies of the K35 amino acid substitution (“1”) were as effective as a homozygous *TMEM154* “knockout” with the frame-shift deletion mutation (“4”) in reducing infection susceptibility. When considering ewe reproductive performance, a difference was not detected when comparing haplotypes “2”, and “3” to each other, or “1” and “4” to each other. Our study indicated that ewes with two copies of the severely truncated versions of *TMEM154* (“4,4”) had normal lamb productivity. Without complete understanding of the natural function of *TMEM154* our recommendations to producers interested in using *TMEM154* selection to reduce their flock’s genetic predisposition to OPP are encouraged to increase the frequency of *TMEM154* haplotype K35 (“1”) since it encodes a full-length protein with minimal difference to the ancestral polypeptide.

## 1. Introduction

Visna/Maedi virus (VMV) and its closely related North American counterpart, Ovine Progressive Pneumonia Virus (OPPV) produce a lymphoproliferative disease which is invariably fatal. Although the disease affects multiple organs including the central nervous system, mammary glands, and carpals, it is the progressive lung infection with interstitial pneumonia that is most commonly debilitating. In the U.S., the disease (OPP) causes extensive financial losses due to a decrease in ewe productivity of about 20% and eventual culling or death of breeding stock [1,2]. Seroprevalence studies of U.S. sheep have shown that 36% of sheep operations have infected animals and 24% of all animals tested were seropositive [3]. Once infected, sheep remain infected for life, and there is no effective treatment or vaccine. OPP can be eradicated from a flock by testing and simultaneous removal of infected sheep or by testing and isolation of all young ewe lambs and isolating uninfected replacement ewes from the infected flock. While effective, these management procedures are often laborious, expensive, and do little to improve genetic resistance to OPP. Thus, OPP-free flocks may remain genetically predisposed to OPPV infection and disease progression if subsequently exposed to other infected sheep.

Aretrospective matched case–control study with 50,000 genome-wide single nucleotide polymorphism (SNP) markers was conducted previously. In that study host genetic variation within the ovine gene *TMEM154* was associated with reduced susceptibility to infection by OPPV [4]. The function of this type I transmembrane protein is unknown in any species. This gene encodes multiple missense and frameshift variants that comprise more than a dozen distinct polypeptide haplotypes, some of which are associated with reduced susceptibility to infection. The list of variant amino acids encoded by *TMEM154* include: R4A(Δ53), P7H, A13V, L14H, T25I, E31Q, D33N, E35K, G38R, T44M, N70I, I74F/V, E82Y(Δ 82), I102T, and I105V [5,6,7]. Some combinations of these coding variants are presumed to affect *TMEM154* function and consequently OPPV infection. This large number of coding variants presents a special challenge for genotype analysis since multiple combinations of allelic variants could occur on the same molecule of DNA and a single non-synonymous SNP variant is not sufficient to uniquely identify a polypeptide isoform. For example, the E35 residue is encoded on haplotypes with both high and low susceptibility. Thus, identification of the polypeptide isoform encoded by the *TMEM154* haplotype is essential for correctly evaluating genetic association.

The four most common *TMEM154* haplotypes overall in US sheep were designated “1” through “4” and represent 97% of those tested [5]. The ancestral haplotype “3” encodes these residues: R4, P7, A13, L14, T25, E31, D33, E35, G38, T44, N70, I74, E82, I102, and I105). Haplotypes “1”, “2”, and “4” are each identical to “3” except for these residues: K35, I70, and A4 Δ53/M44; respectively. The occurrence of one or two copies of either haplotypes “2” or “3” is associated with increased susceptibility to OPPV infection. Conversely, homozygosity of haplotype “1” is associated with decreased susceptibility to OPPV infection. The deletion haplotype “4” typically has a frequency less than 5% in sheep populations and has not been rigorously tested for association with infection. However, these association with haplotypes “1”, “2”, and “3” have been reproduced around the world with different sheep populations, viruses, and management environments [6,7,8,9,10,11,12,13]. While other sheep variants have been reported to be associated with OPP, replication and validation in prospective challenge studies is lacking [14,15,16,17,18,19].

A weakness in many published genetic association studies measuring OPPV susceptibility is the lack of consistent multiyear natural virus exposure with serial sampling, use of consistent genetic background, and a failure to account for full haplotypes. Here, we present a longitudinal study, where three experimental populations of sheep were produced with common overall genetic backgrounds within experimental flocks while specifically segregating combinations of two *TMEM154* haplotypes alleles each (e.g., “2” versus “3”, and “1” versus “4”). Our aim in this effort was to provide an unbiased evaluation of the relative impacts of the four most common haplotypes present in U.S. sheep with haplotype “4” representing a naturally occurring knock-out allele. Repeated serological evaluation was conducted every four months over the course of five years of natural virus exposure to estimate direct allele effects and gene action. Evaluation of susceptibility and ewe productivity from the first experimental population (haplotype “1” versus “3”) has been reported previously [7,8,20] and is only included for completeness and as the source of continued virus exposure (Table 1). The focus of the current manuscript is thus on the second and third of these experimental flocks. Our results here provide the most complete and novel investigation into the allele effects, gene action, and estimated impact on susceptibility to infection and total ewe productivity for all four common *TMEM154* haplotypes present.

## 2. Results

### 2.1. Description of Experimental Flocks

Experimental flock 1 represents the evaluation of susceptibility and ewe productivity from the first experimental population (haplotype “1” versus “3”) and has been reported previously [7,8,20]. It is included here for completeness and represents the source of continued virus exposure (Table 1). Results are presented separately for Experimental flocks 2 and 3 as each has a different average genetic background for performance traits. Experimental flock 2 is ½ Romanov and thus has a higher reproductive rate than ewes in Experimental flock 3 which is ½ Katahdin and ½ Rambouillet. Additionally, lifetime performance values were for 5 years in Experimental flock 2 and 4 years for Experimental flock 3. Inferences should only be drawn between diplotypes (diploid combination of two haplotypes) within an Experimental flock. Within a flock, each diplotype is represented by the same average genetic background, and inferences can be made between diplotypes.

### 2.2. Serological OPPV Infection Status

#### 2.2.1. Experimental Flock 2

**Figure 1:**
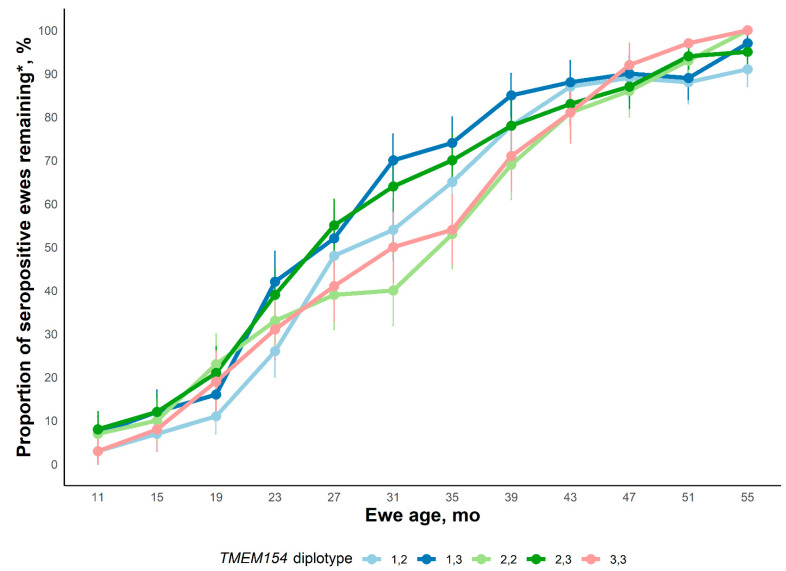
Proportion of ewes in Experimental flock 2 that were serologically positive by age of ewe. Ewes were sampled every 4 months throughout the experiment until three positive tests had been accumulated. Average least square means are presented at approximately each sampling age. The interaction of Age x Diplotype was not significant (*p* = 0.94). The main effect of Diplotype was also not significant (*p* = 0.84). The main effect of ewe age was significant (*p* < 0.001). * Since ewes were culled from the flock for welfare reasons, the apparent seroprevalence can decrease on a percentage basis within a Diplotype group between time points measured.

The full model considering OPPV infection status through 67 mo of age would not converge, most likely because of few observations and little variation (i.e., most ewes were seropositive). To achieve convergence, serology data taken after 55 mo of age was excluded and least-square means for the ewe age x *TMEM154* diplotype interaction effect on OPPV infection status are displayed in Figure 1. Neither the ewe age x *TMEM154* diplotype interaction effect nor the main effect of *TMEM154* diplotype impacted OPPV infection status (*p* ≥ 0.75). However, the main effect of ewe age greatly influenced OPPV infection status (*p* < 0.001) and, by 43 mo, >80% of ewes were seropositive. At 67 mo of age (data not shown), 21/23, 31/32, 18/18, 26/28, and 15/15 of the remaining *TMEM154* diplotype “1,2”; “1,3”; “2,2”; “2,3”; and “3,3” ewes were OPPV seropositive, respectfully. This result indicates that haplotype “2” and haplotype “3” are both equally dominant to haplotype “1” thus representing similar levels of risk for infection by OPPV.

#### 2.2.2. Experimental Flock 3

**Figure 2:**
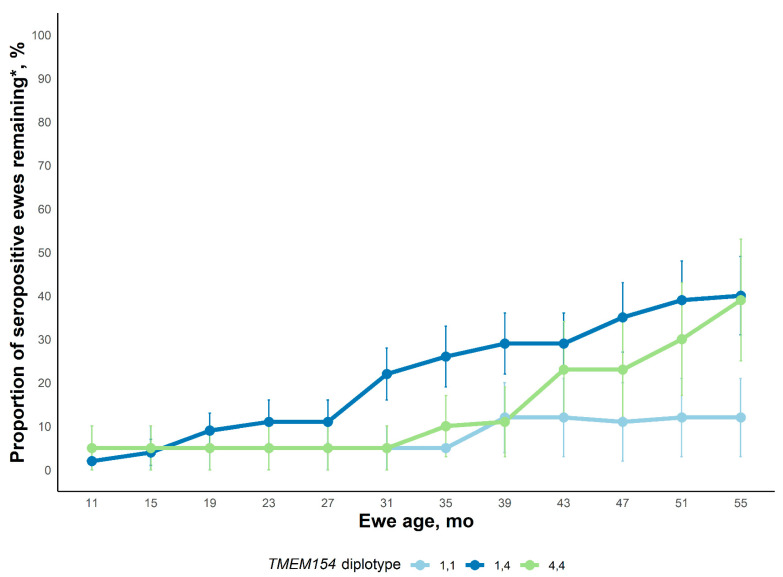
Proportion of ewes in Experimental flock 3 that were serologically positive by age of ewe. Ewes were sampled every 4 months throughout the experiment until three positive tests had been accumulated. Average least square means are presented at approximately each sampling age. The interaction of Age x Diplotype was not significant (*p* = 0.90). The main effect of Diplotype was also not significant (*p* = 0.53). The main effect of ewe age was significant (*p* < 0.001). * Since ewes were culled from the flock for welfare reasons, the apparent seroprevalence can decrease on a percentage basis within a diplotype group between time points measured.

Serological status of ewes by diplotype within Experimental flock 3 over time is presented in Figure 2. Least-square means for the ewe age x *TMEM154* diplotype interaction effect on ewe OPPV infection status are displayed. Ewe seropositivity was not impacted by the age x *TMEM154* diplotype interaction effect or the main effect of *TMEM154* diplotype (*p* ≥ 0.53) but did gradually increase with age (*p* < 0.001) indicating that all combinations of *TMEM154* diplotypes can become infected given enough exposure and time. At 55 mo of age, 2/11, 10/35, and 6/13 of the remaining *TMEM154* diplotype “1,1”; “1,4”; and “4,4” ewes were seropositive, respectively. At the end of the evaluation only 10% of the diplotype “1,1” ewes were serologically positive compared to roughly 38% of the diplotype “1,4” or “4,4” ewes. The natural fallout of ewes by 55 mo of age that happens in any population regardless of genetic value did not allow us to detect this difference between those diplotypes as significant.

### 2.3. Annual Reproductive Performance

The main effect of birth year and related two-way interactions were included in all ewe performance models but are not presented or discussed beyond significance since birth year effects cannot be predicted to recur in the future.

#### 2.3.1. Experimental Flock 2

Least-squares means for the main effects of ewe age and *TMEM154* diplotype on annual performance traits are displayed in Table 2. The birth year of the ewe x age interaction effect was significant (*p* < 0.01) for all annual performance traits, but no other two-way interactions were (*p* ≥ 0.06). Ewe body weight (BW) at mating increased with age and was different between each age class (*p* < 0.001). Three-, 4-, and 5-yr-old ewes had the greatest number lambs born (NLB), 2-yr-olds were intermediate, and 1-yr-olds the least (*p* < 0.001). One- and 5-yr-old ewes had similar number lambs weaned (NLW) (*p* = 0.55), but litter wean weight (LWW) was greater for 5- than 1-yr-old ewes (*p* < 0.001) and 2-, 3-, and 4-yr-old ewes were higher performing for both traits (*p* < 0.001). The main effect of *TMEM154* diplotype did not impact any annually recorded ewe trait (*p* > 0.10).

#### 2.3.2. Experimental Flock 3

Least-squares means for the main effects of ewe age and *TMEM154* diplotype on annual performance traits are displayed in Table 3. The birth year of the ewe x age interaction effect was significant (*p* < 0.01) for BW and NLB, but no other two-way interactions were significant for annual performance traits (*p* ≥ 0.08). Ewe BW at mating increased with age and was different between each (*p* < 0.001). Three- and 4-yr-old ewes had the greatest NLB, 2-yr-olds were intermediate, and 1-yr-olds the least (*p* ≤ 0.03) whereas only 1-yr-old NLW and LWW was less than other ages (*p* < 0.001). The main effect of *TMEM154* diplotype did not impact any annually recorded ewe trait (*p* > 0.38).

### 2.4. Lifetime Reproductive Performance and Longevity

#### 2.4.1. Experimental Flock 2

Birth year of the ewe impacted total number of lambs weaned per ewe exposed over 5 yr (NLWT) and total weight of lamb weaned per ewe exposed over 5 yr (LWWT) (*p* < 0.001) but no other model effects were significant in the analysis of lifetime performance traits (*p* ≥ 0.15), and least-squares means for *TMEM154* diplotypes are displayed in Table 4. Results indicate that each of the five diplotype combinations was similar in lifetime performance and longevity reinforcing the concept that the “2” and the “3” haplotypes are equivalent risk factor alleles.

#### 2.4.2. Experimental Flock 3

No model effects were significant in the analysis of NLWT, LWWT, or longevity (*p* ≥ 0.23), and least-squares means for *TMEM154* diplotypes are displayed in Table 5. Results indicate that each of the three diplotype combinations was similar in lifetime performance and longevity. The interpretation is that haplotype “1” and haplotype “4” will confer similar levels of reduced susceptibility and lifetime performance. It should be noted that no evidence was detected to indicate that the “4,4” diplotype ewes were impacted by the total loss of function of *TMEM154* since they are a naturally occurring knockout mutation. However, the true function of *TMEM154* in mammals is not yet understood and there may be phenotypic differences in the functional knockout animals that we did not observe or document.

### 2.5. Evidence of Lack of Disease Progression in “4,4” Diplotype Ewes

**Figure 3:**
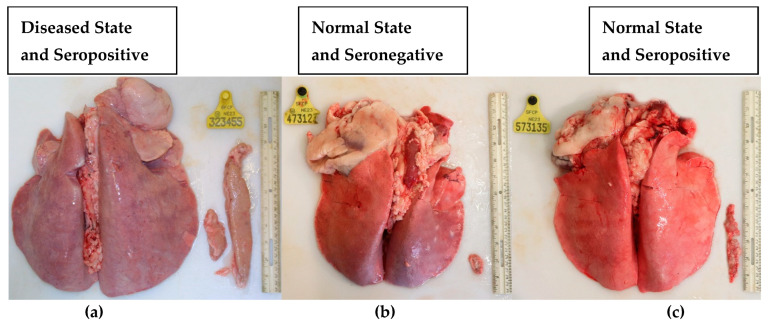
Comparison of representative tissues from a seropositive OPPV infected sheep with that of two “4,4” diplotype ewes one seronegative and one seropositive for OPPV. (**a**) Lungs and mediastinal lymph nodes of a seropositive sheep with lesions typical for OPP. The lungs are mottled grey and the mediastinal lymph nodes are greatly enlarged. Histopathology indicated hallmark severe lymphoproliferative inflammation. (**b**) Tissues from a 6-year-old “4,4” *TMEM154* diplotype ewe that was serologically negative for OPPV at the end of the evaluation experiment. Both lung and mediastinal lymph tissues were normal in size and appearance by digital palpation. The mild red discoloration of the caudodorsal aspects of the lungs is caused by how they were positioned post-mortem. Any discoloration would be best described as dependent passive hemostatic congestion. (**c**) Tissues from a 5-year-old “4,4” *TMEM154* diplotype ewe that was serologically positive for OPPV by 17 months of age. At the time of tissue collection, this ewe had been serologically positive for a minimum of 40 months. Both lung and mediastinal lymph tissues were normal in appearance and by digital palpation.

After the experiment was completed, there were eight “4,4” ewes available for necropsy. Four of these ewes had been seropositive for 21–40 months while the other four had remained seronegative. All eight ewes were euthanizedon a single day and the lungs and mediastinal lymph nodes were evaluated for signs of subclinical OPP. All lung tissues and mediastinal lymph nodes from all OPPV seropositive ewes were indistinguishable from seronegative ewes and appeared normal and healthy. Figure 3 shows typical lung and lymph node lesions of a seropositive sheep with advanced OPPV infection in comparison to two representative “4,4” diplotype ewes one of which was seropositive and the other seronegative. Although, similar necropsies were not performed on any “1,1” and “1,4”, these results indicate that seropositivity does not always predict subclinical progression of disease.

## 3. Discussion

This manuscript describes estimates of allele effects and gene action among the four most common ovine *TMEM154* haplotypes for OPPV risk and their impact on annual and life-time ewe productivity. The highly susceptible haplotypes “2” and “3” differ by only a single amino acid (N70I) yet were equivalent dominant risk factors for OPPV infection reaching nearly 100% infection levels in 55 months. Additionally, there were no indications that either haplotype “2” or “3” contributed novel, beneficial reproductive performance traits that would justify their retention in OPP-affected flocks. The reduced susceptibility haplotypes “1” and “4” were very similar in their effects yet are completely different in their sequences. Haplotype “1” differs by only one amino acid compared to the ancestral haplotype (E35K), while the frameshifted haplotype “4” shares only the first three 3 of 191 residues and is truncated after 57 residues compared to the ancestral haplotype. Despite these differences, haplotypes “1” and “4” in any combination reduced the risk of OPPV infection by similar amounts. The seropositive proportion of “1,1”; “1,4” and “4,4” ewes was statistically negligible (10% to 40%) after 55 months of exposure and neither diplotype combination affected ewe reproduction in a negative way. This indicates two important outcomes: first, the K35 *TMEM154* variant has the same effect on infection risk as knocking out the whole gene, and second, knocking out *TMEM154* (e.g., “4,4” ewes) had no effect on any obvious phenotypic difference in growth and development or in reproductive rates or lifetime performance. These results provide key information for developing effective selection strategies in sheep industries affected by ovine lentivirus diseases.

The prospective cohort study results present here were entirely consistent with previous reports of retrospective matched cases control studies [4,13] and related prospective cohort studies [6,7,8,9,10,11,12,20]. Ewes with two copies of haplotype “1” show significantly reduced susceptibility to lentivirus infection compared to those with one or two copies of haplotypes “2” or “3” reported. The first phase of our five-year prospective cohort study compared only haplotypes “1”and “3” [7,8,20]. At 9 mo of age, predicted probabilities of infection for diplotype “1,1”; “1,3”; and “3,3” ewe and wether lambs were 0.09, 0.32, and 0.35, respectively [7]. Ewe lambs were retained and predicted probabilities of infection at 39 mo of age for diplotypes “1,1”; “1,3”; and “3,3” were 0.10, 0.88, and 0.89, respectively [8]. Average lifetime ewe productivity was also accumulated as diplotype “1,1” ewes weaned, on average, 2.1 more lambs and 40 kg greater weight of lamb over 5.5 production year than diplotype “1,3” and “3,3” ewes [20]. To date there have been no other reports of prospective natural challenge studies involving *TMEM154* in any species. Using economic values at the time of the experiment indicated these differences amounted to an increased value of at least US $171 for each “1,1” diplotype ewe compared to “1,3” or “3,3” diplotypes.

The relatively rare *TMEM154* “knockout” diplotype “4,4” has attracted attention since it was first reported in 2012 since it offered the possibility of complete resistance [4]. However, in 2015 OPP positive sheep with the “4,4” diplotype were identified and viral genomes were isolated [21]. The lack of disease progression among seropositive “4,4” diplotype ewes suggest that animals with the least susceptible diplotype combinations may have limited viral replication. This is consistent with a recent report of a “1,1” diplotype ram that maintained very low viral loads and ultimately apparently cleared infection to below detectable limits [22]. This phenomenon may be a possible explanation for inconsistent report of performance levels of serologically positive sheep compared to serologically negative sheep when using point in time surveys [1,2,23,24].

One weakness of this study was the limited number of ewes used for comparing effects of 1,1 and 4,4 diplotypes. This was due to the use of only females for the longitudinal study and having to first produce a uniform F1 population of “1,4” sires and dams a priori. However, this was balanced by a strength of the study: comparisons in ewes from a uniform genetic background. A second weakness of the study was using serostatus as a proxy for infection. Recent reports of cleared VMV infections [22], and the lack of disease progression in ewes with “4,4” diplotypes now raise the possibility that ewes with “1,1”, “1,4” and “4,4” diplotypes can clear infections. A third weakness was necropsy of only “4,4” diplotype sheep to test disease progression. The original design did not included necropsy of ewes to collect lung tissues. In hindsight, it would have been informative to have collected tissues from all diplotype combinations at 55 months. In addition to uniform genetic backgrounds of ewes, other strengths of this study included, serially blood sampling to reduce the variability associated with ELISA-based testing procedures, and the five-year exposure time with at least 30% infected flock mates.

Genetic selection programs in U.S. sheep are becoming more advanced with the use of genome assisted breeding values, combining whole genome SNP panels with performance recording. The US National Sheep Improvement Program (NSIP) is currently developing a genetic component where breeders will have the future option to use *TMEM154* haplotype information from their SNP genotyping program. Thus, based on results of the present study we offer three recommendations to producers affected by OPPV or at risk for future OPPV exposure concerning *TMEM154* variants:

- decrease the frequency of haplotypes “2” and “3”
- increase the frequency of haplotype “1”
- increase the frequency of haplotype “4” at the expense of haplotypes “2” and “3” but not at the expense of haplotype “1”

The rationale for the last recommendation is that haplotype “1” (K35) was as effective as haplotype “4” (the knockout), yet only one amino acid different from the ancestral protein sequence. Thus, haplotype “1” has the best chance at retaining the natural function of the *TMEM154* gene, which is presently unknown.

## 4. Materials and Methods

### 4.1. Diplotype Determination

*TMEM154* genotyping was performed at Neogen (Lincoln, NE) with Matrix-assisted laser desorption/ionization-time of flight (MALDI-TOF) mass spectrometry (MS) according to the manufacturer’s instructions (Agena Biosciencce, San Diego, CA). Briefly, multiplex assays were designed with commercial software and adjusted manually. Haplotype “3” is the ancestral haplotype encoding glutamate (E) at position 35, and asparagine (N) at position 70. Haplotypes 2 differs from this ancestral haplotype by a single amino acid substitution of Isoleucine (I) at position 70. The key feature of haplotype 1 is the presence of lysine (K) residue at position 35. Whereas haplotypes 1, 2 and 3 encode full-length polypeptides, haplotype 4 has a frameshift deletion (delta53) that is predicted to cause premature termination of translation and loss of protein function from codon 4 on. Haplotype 4 is relatively rare, with a frequency less than 2% in U.S. populations. The occurrence of methionine residue at position 44 has also been used to infer haplotype 4 by assuming complete linkage disequilibrium with A4(delta53). This assumption is based on the observation that an A4(delta53) allele was invariantly present with an T44 allele in more than 8000 U.S. sheep previously genotyped by Sanger sequencing at USMARC [5].

### 4.2. Creation of Segregating Experimental Ewe Flocks

#### 4.2.1. Experimental Flock 1

To produce a suitable population for testing additive and dominance effects of haplotypes “1” and “3”, twelve Composite IV (1/2 Romanov, ¼ White Dorper, ¼ Katahdin) rams of diplotype “1,3” were mated to ½ Romanov ½ Rambouillet ewes that were also diplotype “1,3” and were all serologically positive for OPP serving as the initial source of infection for the remaining overlapping populations. Results of this Experimental flock 1 were previously published (7–8,20).

#### 4.2.2. Experimental Flock 2

To produce a suitable population for testing additive and dominance effects of haplotypes “2” and “3”, in 2010 Romanov rams of diplotype “2,3” were mated to Rambouillet ewes, with predominantly diplotype “1,1”. Of the resulting Romanov x Rambouillet ewe lambs (frequencies were 55, 59, 13, and 12 for diplotypes “1,2”, “1,3”, “2,3”, and “3,3”, respectively) 139 were kept for breeding in 2011 to five Composite IV (1/2 Romanov, ¼ White Dorper, ¼ Katahdin) rams that were diplotype “2,3”. These matings were repeated the next two years. Thus, Experimental flock 2 evaluation ewes were born in years 2012, 2013, and 2014. Ewe productivity through five years of age was evaluated from 2013 to 2019.

#### 4.2.3. Experiment Flock 3

To produce a suitable population for testing additive and dominance effects of haplotypes “1” and “4”, two Katahdin rams homozygous diplotype “4,4” and one Katahdin ram heterozygous diplotype “1,4” were mated to 186 Rambouillet ewes predominantly diplotype “1,1” in 2010. Progeny of both sexes from these matings that were diplotype “1,4” were *inter se* mated to produce Experimental flock 3 ewe lambs born in years 2013, 2014, and 2015. All ewe lambs were ½ Katahdin and ½ Rambouillet. Ewe productivity through four years of age was evaluated from 2014 to 2019.

### 4.3. Flock Husbandry and Ewe Performance

All ewes were exposed annually to Dorset rams in multi-sire mating groups in late September through early October. Dorset rams were used to ensure all progeny exhibited 100% individual heterosis. Ewe to ram ratios at breeding averaged 20.7 ewes per ram (range 14.5 to 26.8). The exception to this generalization was in the last two years of the evaluation is that some ewes (*n* = 9) that would normally have been culled and disposed were maintained with the ewe flock as a source of virus exposure but were not allowed to be exposed to rams and contribute to ewe productivity data. All three Experimental flocks were managed as one contiguous group with an OPPV infection rate > 30% and direct or fence line contact throughout the production year. Flock health was monitored according to USMARC standard operating procedures and included annual vaccinations (Campylobacter jejuni/fetus, Chlamydia psittaci, and Clostridia) and, when necessary, chemical treatment for internal and external parasites. Approximately 1 mo before expected lambing date each year, ewes were shorn and managed in drylot with access to an open-fronted pole barn until lamb weaning. Ewes were fed a corn-silage based ration while in semi-confinement (breeding and late gestation through lactation) and rotated through actively growing or stockpiled forage for the remainder of the production year. After lambing, ewes and newborn lambs were placed into individual bonding pens for approximately 24 h before joining larger contemporary groups. First parity ewes were permitted to rear a maximum of two lambs, whereas second parity and older ewes were permitted to rear up to three lambs. No grafting took place and excess lambs or those born to dams that had died or had insufficient milk production were transferred to the nursery for artificial rearing until 4 wk of age. Dam-reared lambs had access to supplemental feed while still nursing and were weaned at 8 wk of age.

Annually recorded ewe traits included body weight (BW) at mating and number of lambs born (NLB) and weaned (NLW) per ewe exposed. Additionally, lamb BW at birth was used to adjust BW at weaning to 65 d of age which was summed within dam to calculate total litter weaning weight per ewe exposed (LWW). Ewes were not credited with nursery-reared lamb performance in NLW or LWW calculations. The experiment was initially designed to assess lifetime performance over five parities, but the experiment was truncated by one year due to concerns about overall consistency of virus exposure and to adjust for other research priorities using our facilities. This resulted in Experimental flock 3 termination one year earlier than designed and, therefore, 2015-born ewes were not exposed for a 5th parity. Longevity was expressed as absence or presence in the flock at the end of the experiment (0 or 1, respectively) at 67 mo (Experimental flock 2) or 55 mo of age (Experimental flock 3). Lifetime productivity was assessed as total number (NLWT) and weight (LWWT) of lamb weaned through five (Experimental flock 2) or four production years (Experimental flock 3).

### 4.4. Serological Monitoring

Blood samples were collected from ewes at approximately 4 mo intervals coinciding with breeding (September through October), late pregnancy (January through February), and lamb weaning (May through June). Ewes were bled by jugular venipuncture using 9-mL S-Monovette serum Z syringes (Sarstedt, Newton, NC, USA). Bleeding took place on up to 15 dates from 11 mo through 67 mo of age in Experimental flock 2 and on up to 12 dates from 11 mo through 55 mo of age in Experimental flock 3. Competitive ELISA tests (VMRD Inc., Pullman, WA) were used for serological testing of serum samples at either USMARC or Neogen (Lincoln, NE, USA). The threshold value for percentage inhibition used to signify positivity of a single test was 35% based on the manufacturer’s recommendation. Three sequential positive tests over 12 months were required to confirm infection but were retrospectively considered infected at the first date of three repeated positive tests. Once an individual ewe had tested positive three times, it was inferred to be positive for the remainder of the experimental evaluation without re-testing.

### 4.5. Tissue Collection in “4,4” Diplotype Ewes

Due to the low frequency of TMEM154 “4,4” diplotypes in most sheep populations, disease progression of this natural knockout is difficult to assess. However, at the end of our experiment we had eight “4,4” ewes available for necropsy. Four of these ewes were serologically positive for OPPV for 21–40 months, while four ewes remained serologically negative throughout the experiment. The IACUC protocols were thus amended and approved for necropsy examination and tissue collection of these animals. Tissues were collected and used for several purposes, including: 1) sequencing of the virus strain infecting these animals (lung and lymph tissue), 2) comparing the gross morphology of the lungs, mediastinal lymph node, and heart to previous OPP cases in animals of various genotypes, and 3) optimizing conditions to culture blood monocytes to stimulate virus production for future virus sequencing efforts. The ewes were euthanized and the lungs and mediastinal lymph nodes were evaluated for indications of typical characteristics associated with OPP.

### 4.6. Statistical Analysis

All statistical analyses were conducted in the GLIMMIX procedure of SAS (v 9.4; SAS Institute Inc., Cary, NC, USA). Annually recorded performance traits (BW, NLB, NLW, and LWW) were analyzed as repeated measures with fixed effects of birth year of the ewe (Experimental flock 2: 2012–2014; Experimental flock 3: 2013–2015), age (Experimental flock 2: 1–5 yr; Experimental flock 3: 1–4 yr), and *TMEM154* diplotype (Experimental flock 2: “1,2”; “1,3”; “2,2”; “2,3”; or “3,3”; Experimental flock 3: “1,1”; “1,4”; or “4,4”). Additionally, a random sire of the ewe effect (Experimental flock 2: *n* = 5; Experimental flock 3: *n* = 12) was fit and a compound symmetric (co)variance structure with heterogenous variance across age was chosen to model the random ewe effect. Lifetime performance traits (longevity, NLWT, and LWWT) were analyzed with fixed effects of birth year of the ewe and *TMEM154* diplotype and a random effect of sire of the ewe. Ewe OPPV infection status was analyzed as repeated measures with fixed effects of ewe age in nearest month (Experimental flock 2: up to 15 timepoints; Experimental flock 3: up to 12 timepoints) and *TMEM154* diplotype and the random ewe effect was modeled with a compound symmetric (co)variance structure with heterogenous variance across age. All two-way interactions among fixed effects were included in these analyses.

Ewe longevity and OPPV infection status were modeled as binary traits (logit link function) and means were back-transformed to the original scale. All other traits were considered normally distributed. Records from ewes culled from the flock as “surplus” were not included in the analysis of lifetime performance but were for annual performance and OPPV infection status. Therefore, number of ewes with *TMEM154* diplotypes “1,1”; “1,4”; and “4,4” were, 28, 57, and 24, respectively, for annual performance traits and OPP infection status. For lifetime performance traits there was one less “1,4” ewe.

## 5. Conclusions

The relative impact of the four most common ovine *TMEM154* variants on lifetime susceptibility to OPPV infection and ewe performance was significant. When persistently exposed to natural virus challenge over five years, the highly susceptible haplotypes “2” (I70) and “3” (ancestral) had equal and complete dominance over the reduced susceptibility haplotype “1” (K35). This resulted in more than 80% infection of ewes that were diplotypes “1,3”,”1,2”, “2,2”, “2,3”and “3,3” by 3.3 yr of age, compared to less than < 10% of diplotype “1,1” ewes that became infected through 5.5 yr of age. In addition, the least susceptible diplotype combinations “1,1”, “1,4” and “4,4” (knockout), were similarly protective and ewes with these diplotypes had similar levels of reproductive performance. Recommendations for haplotype use included: increasing the frequency of “1” and “4” at the expense of “2” and “3”; and increasing “1” at the expense of “4”.

## Figures and Tables

**Table 1:** Description of the three overlapping experimental evaluation ewe flocks.

Experimental Flock ^1^	*TMEM154* Diplotypes ^2^	Breed Composition ^3^	Birth Years	Evaluation Years
1 (*n* = 108) see [7,8,20]	“1,1”, “1,3”, “3,3”	½ RV, ¼ RB, 1/8 WD, 1/8 KT	2011	2012–2016
2 (*n* = 269)	“1,2”, “1,3”, “2,2”, “2,3”, “3,3”	½ RV, ¼ RB, 1/8 WD, 1/8 KT	2012–2014	2013–2019
3 (*n* = 114)	“1,1”, “1,4”, “4,4”	½ RB, ½ KT	2013–2015	2014–2019

^1^ Number of unique ewes present at beginning of experimental evaluation first mating season.^2^ The diploid combination of two haplotypes.^3^ RV = Romanov, RB = Rambouillet, WD = White Dorper, KT = Katahdin

**Table 2:** Least-squares means (± SE) for the main effects of ewe age and *TMEM154* diplotype on annual body weight at breeding and reproductive performance in Experimental flock 2.

		Trait ^1^			
Effect	Level	BW, kg	NLB, n	NLW, n	LWW, kg
Age, yr	1	39.5 ± 0.42 ^e^	1.39 ± 0.06 ^c^	1.01 ± 0.06 ^b^	16.3 ± 0.92 ^c^
	2	51.0 ± 0.49 ^d^	2.06 ± 0.07 ^b^	1.61 ± 0.06 ^a^	27.6 ± 1.06 ^a^
	3	58.5 ± 0.52 ^c^	2.32 ± 0.07 ^a^	1.68 ± 0.07 ^a^	29.4 ± 1.11 ^a^
	4	63.6 ± 0.54 ^b^	2.36 ± 0.08 ^a^	1.50 ± 0.08 ^a^	27.4 ± 1.42 ^a^
	5	65.2 ± 0.64 ^a^	2.41 ± 0.09 ^a^	1.13 ± 0.08 ^b^	21.7 ± 1.52 ^b^
*TMEM154 diplotype*	“1,2”	55.5 ± 0.74	2.15 ± 0.08	1.43 ± 0.07	25.6 ± 1.43
	“1,3”	54.9 ± 0.78	2.09 ± 0.09	1.38 ± 0.08	25.0 ± 1.48
	“2,2”	57.3 ± 0.83	2.11 ± 0.09	1.31 ± 0.08	23.1 ± 1.58
	“2,3”	55.6 ± 0.71	2.06 ± 0.08	1.41 ± 0.07	25.0 ± 1.36
	“3,3”	54.4 ± 0.87	2.12 ± 0.10	1.39 ± 0.09	23.7 ± 1.67

^1^ BW = ewe body weight, NLB = number of lambs born per ewe exposed per year, NLW = number of lambs weaned per ewe exposed per year, LWW = total weight of lamb weaned per ewe exposed per year. ^a–e^ Means within an effect with no common superscript that are different (*p* < 0.001).

**Table 3:** Least-squares means (± SE) for the main effects of ewe age and *TMEM154* diplotype on annual body weight at breeding and reproductive performance in Experimental flock 3.

		Trait ^1^			
Effect	Level	BW, kg	NLB, n	NLW, n	LWW, kg
Age, yr	1	40.4 ± 0.64 ^d^	0.77 ± 0.08 ^c^	0.57 ± 0.08 ^b^	11.3 ± 1.52 ^b^
	2	55.7 ± 1.08 ^c^	1.35 ± 0.10 ^b^	1.10 ± 0.10 ^a^	22.9 ± 2.02 ^a^
	3	66.4 ± 0.97 ^b^	1.71 ± 0.11 ^a^	1.29 ± 0.11 ^a^	27.7 ± 2.23 ^a^
	4	72.2 ± 1.09 ^a^	1.83 ± 0.12 ^a^	1.26 ± 0.12 ^a^	27.9 ± 2.49 ^a^
*TMEM154 diplotype*	“1,1”	58.2 ± 1.24	1.38 ± 0.13	1.02 ± 0.11	21.3 ± 2.41
	“1,4”	58.1 ± 0.90	1.49 ± 0.09	1.13 ± 0.08	24.6 ± 1.75
	“4,4”	59.7 ± 1.29	1.37 ± 0.13	1.02 ± 0.12	21.6 ± 2.44

^1^ BW = ewe body weight, NLB = number of lambs born per ewe exposed per year, NLW = number of lambs weaned per ewe exposed per year, LWW = total weight of lamb weaned per ewe exposed per year. ^a–d^ Means within an effect with no common superscript are different (*p* ≤ 0.03).

**Table 4:** Least-square means (± SE) for the main effect of *TMEM154* diplotype on reproductive performance and longevity through five production years in Experimental flock 2.

	Trait ^1^		
*TMEM154 diplotype*	NLWT, n	LWWT, kg	Longevity, p
“1,2”	5.80 ± 0.42	102.7 ± 8.39	0.48 ± 0.08
“1,3”	5.61 ± 0.45	102.5 ± 8.89	0.60 ± 0.08
“2,2”	5.26 ± 0.48	92.7 ± 9.37	0.51 ± 0.09
“2,3”	5.46 ± 0.40	97.8 ± 7.94	0.53 ± 0.07
“3,3”	5.45 ± 0.50	94.4 ± 9.82	0.54 ± 0.10

^1^ NLWT = total number of lambs weaned per ewe exposed over 5 yr, LWWT = total weight of lamb weaned per ewe exposed over 5 yr, Longevity = absence or presence (0 or 1, respectively) after 5 yr.

**Table 5:** Least-square means (± SE) for the main effect of *TMEM154* diplotype on reproductive performance and longevity through four production years in Experimental flock 3.

	Trait ^1^		
*TMEM154 diplotype*	NLWT, n	LWWT, kg	Longevity, *p*
“1,1”	2.82 ± 0.55	59.8 ± 12.0	0.41 ± 0.12
“1,4”	3.68 ± 0.45	79.5 ± 9.76	0.63 ± 0.09
“4,4”	3.35 ± 0.61	71.0 ± 13.3	0.63 ± 0.14

^1^ NLWT = total number of lambs weaned per ewe exposed over 4 yr, LWWT = total weight of lamb weaned per ewe exposed over 4 yr, Longevity = absence or presence (0 or 1, respectively) after 4 yr.

## Data Availability

The data presented in this study are available on request from the corresponding author.
